# Ciliated Epibionts Modify the Cardiac Stress Reaction to Perceived Predation in Daphnia

**DOI:** 10.3390/microorganisms12061219

**Published:** 2024-06-18

**Authors:** Andrew K. Davis, Helen Gloege

**Affiliations:** 1 Odum School of Ecology, University of Georgia, Athens, GA 30602, USA; 2 Department of Biological Science, Mount Holyoke College, South Hadley, MA 01075, USA

**Keywords:** daphnia, heart rates, acute stress, predation, epibionts, *Vorticella*

## Abstract

When animals perceive an acute stressor like a predator, they typically undergo a suite of physiological changes that function to improve survival during the encounter, such as elevation in cardiac output, to supply more energy to muscles. If bodily energy is limited, such as by parasites or infections, these functions could become less efficient and lessen host survival. In the aquatic world of microorganisms, individuals can become colonized by other organisms on their surface (epibionts), which could sap energy from their host from their weight, or even compete with the host for food. Here, we tested if one epibiont (a ciliated protozoan, *Vorticella* spp.) affects its hosts’ ability to mount a physiological stress reaction. We collected wild daphnia (*Daphnia ambigua*) that had varying burdens of these on their bodies and exposed them to a simulated stressor (crushed daphnia, to simulate nearby predation) under a microscope while monitoring for changes in their heart rates in real time. Out of 121 daphnia, those with no *Vorticella* epibionts showed no meaningful changes in their heart rate after exposure, but those with light or heavy burdens showed immediate elevations (within 5 min). Moreover, the heart rates of heavily burdened daphnia continued to rise for 1.5 h thereafter, to as much as 17% higher than at baseline. These patterns were unexpected, as they suggest that the ciliated epibionts act to elevate their hosts’ physiological reaction, rather than dampen it, perhaps by churning the water column around the host, thereby enhancing the chemical alarm cue. The procedures used in this study may be useful for future investigations into the acute stress reactions of daphnia or other microorganisms.

## 1. Introduction

All animals, from the macro- to the microscopic, become faced with threats or situations in their lives that are stressful, at some point(s) during their lives, be they encounters with predators, fights with rivals, or having to contend with harsh environmental conditions. Most species display behavioral and physiological changes during these bouts of stress that are designed to enhance survival during the encounter, such as improved blood flow to muscles, enhanced reflexes, or upregulation of immune function [1,2,3,4]. Such changes inherently draw upon the host’s energy stores to manifest, meaning that if energy is limited for some other reason, these life-saving bodily processes may not function adequately during that critical time. Importantly, a small body of research from a variety of taxonomic groups is beginning to show how parasites and/or diseases can alter a host’s physiological stress reactions, either to increase the magnitude of the reaction or to limit it. Thus far, these patterns have been found in species of insects [5], amphibians [6], fish [7], and birds [8]. The mechanisms of these patterns likely vary, but may involve the energetic cost of the parasites. Diseases and parasites are well known to lead to lethargy and/or reduced physical movement, likely because energy is being diverted to combat infections. This is true in the world of microorganisms as well; one study revealed how individual water fleas (daphnia) infected with a pathogenic fungus have a 36% lower heart rate than normal [9], indicating a chronic drain on host energy.

Endoparasites are not the only potential drain on host energy, especially in the aquatic realm. Freshwater zooplankton such as daphnia also serve as substrates or attachment surfaces for a wide variety of other microscopic organisms, collectively termed epibionts [10]. These organisms are not necessarily parasitic in nature, although they are sometimes evaluated along with known parasites [11,12]. In fact, they can have a wide range of positive or negative effects on their hosts (i.e., the substrate organism), depending on their nature, and their within-host abundance, as reviewed in [13]. For example, organisms in the genus *Vorticella* are ciliated protozoans that attach to zooplankton (or other water-borne organisms) and use their cilia to siphon water into their opening for filter feeding [14]. These organisms have long been noted and observed in aquatic-based natural history collections [15,16], and, interestingly, there is evidence that they can compete with their daphnia hosts for food, since they each feed by filtering water [17]. Similar ciliated epibionts can also negatively affect host survival, but only during food limitation [18]. In addition, large epibiont burdens on microorganisms can impose an energetic drain by their heaviness and/or drag, and can impede swimming performance [19,20].

Daphnia are a model microorganism to study for a variety of reasons, including for understanding disease and/or parasite transmission in natural populations, e.g., [21,22,23,24], the physiological effects of diseases [9], and the impact of environmental pollutants or chemicals, e.g., [25,26,27,28], to name a few areas of study. Surprisingly, less studied is their physiological responses to threats, or their individual “stress reactions”, such as the physiological changes that occur when they perceive imminent danger. From past work, it seems that daphnia will readily respond to “chemical alarm cues” from crushed conspecifics (simulated predation), or cues from actual predators, in a manner typical of a physiological stress response—they show an increase in respiration [29], and also, a tendency to group together or to seek cover [30]. These behavioral and physiological changes induced by the stressor should be designed to help the animal deal with the stressor; for example, an increase in respiration suggests an elevation in physical exertion. In fact, at least one study empirically showed how exposure to a simulated predation threat in daphnia leads to improved survival during actual predation attempts [31]. This finding in itself highlights the importance for daphnia (like all organisms) to be able to effectively manifest a functional stress response when needed.

The current study aimed to determine what happens when daphnia that are burdened with ciliated epibionts are faced with a perceived threat—are they just as capable of mounting a physiological reaction as those with no such burden? We conducted the study using wild-caught daphnia that had naturally acquired, ciliated epibionts (*Vorticella* sp.). We exposed these daphnia (some with, some without *Vorticella*) to a perceived predation event (chemical cues from crushed daphnia) while monitoring changes in their heart rates in real time, which we used as an index of their physiological stress reaction. Given the assumed energic burden of the epibionts, we had anticipated that they would dampen the physiological reaction to the stressor, but, in fact, we observed the opposite pattern. We discuss the implications of this finding.

## 2. Methods

### 2.1. Study Organisms

Daphnia were collected from a permanent freshwater pond (approximately 30 m wide) at the residence of the author (Davis) in Oconee County, GA, USA, during the months of June and July 2022. This pond contains a year-round population of *D. ambigua* as well as other microorganisms, including Chaoborus larvae (Davis, pers. obs.). Daphnia were collected using a fine-mesh sieve attached to a long pole that was slowly drawn through the water from the bank. The collected daphnia were transported to the lab in containers filled with pond water, and in the lab, the daphnia were maintained in a 10-gallon glass aquarium filled with filtered pond water. In the lab, the room temperature was maintained at 24 °C. We provided supplemental food to the daphnia aquarium each day (spirulina). Since the daphnia were wild, they each had varying degrees of epibiont attachment when we collected them, from none at all to a heavy load (which we quantified after testing). We typically conducted tests (below) on the individual daphnia within 1–3 days of collection. The collections were made weekly during the months of the study.

**Figure 1:**
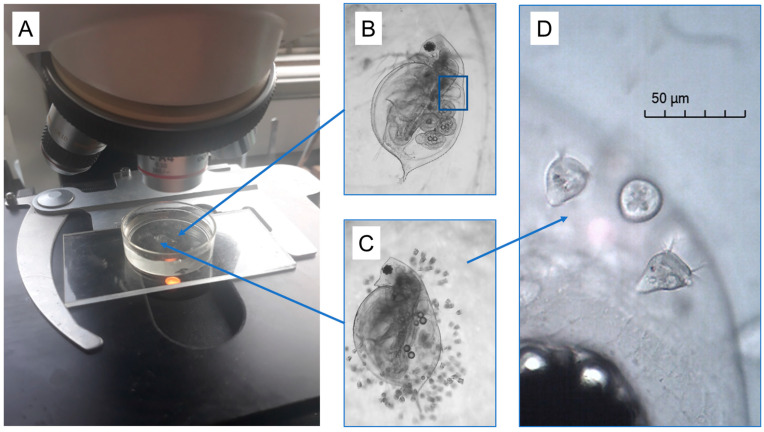
Images of the experimental setup for examining daphnia heart rates before and after the stress exposure. (**A**) Individual daphnia (*D. ambigua*) were gently placed on top of a smear of Vaseline within a 10 mL Petri dish filled with 5 mL of well water. This maintained the animals on their side and in one place, but where they could move their antennae and appendages freely. This allowed us to record video clips of the daphnia heart contractions (blue box) across the 3-h monitoring period, using a camera mounted to a standard light microscope. The daphnia had varying burdens of naturally acquired *Vorticella* epibionts (**B**–**D**). After 60 min, one of us added 0.1 mL of a pre-mixed solution of crushed daphnia (5 animals) to the water to elicit a heart rate elevation.

As noted above, some of the *D. ambigua* in this pond were naturally colonized with varying numbers of ciliated organisms of the genus *Vorticella* (see Figure 1 and Figure S1). Since we were more concerned with the overall burdens of the *Vorticella* in this study, we did not attempt to identify the species itself; there are many similar species in this genus, and, in fact, there are even problems with using morphological characters to differentiate them [14]. We noted there was little variation in *Vorticella* size or appearance across most daphnia, and they tended to attach to the daphnia across their body surface (i.e., they were not clumped; Figure 1C). Upon close inspection at high magnification, we noted that these had the typical *Vorticella* appearance, with a bell-shaped body at the end of a stalk, and with beating cilia surrounding the bell opening. The cilia draw water (and suspended particles) into the bell, where the food is digested.

### 2.2. Stress Procedure

The goal of this procedure was to evaluate the physiological reaction of individual daphnia (with varying epibiont burdens) to a stressful scenario and to be able to monitor their heart reactions to it in real time under a standard light microscope. Thus, our approach was to expose daphnia to doses of crushed conspecifics, which mimics a nearby predation event, and which daphnia can sense via chemical “alarm” cues contained in the crushed material [31]. Prior to each test, we mixed a fresh solution to use for the alarm cue for that test. Using a miniature pestle, we crushed 5 daphnia in a 1.5 mL plastic centrifuge tube that was filled with 0.1 mL of well water.

For the stress procedure itself, we randomly selected and picked up one daphnia from the pondwater container (using a large-bore syringe) and placed it in a 10 mL Petri dish filled with 5 mL of non-chlorinated well water. The daphnia was gently positioned in the middle of the dish and on top of a small smear of Vaseline so that the animal adhered to the smear and on its side. This held the daphnia in place, but allowed full freedom of movement of its antenna and appendages. This is a common approach used in zooplankton investigations where the animal must remain in place but without harm [32]. Other researchers have also used methylcellulose to achieve the same result [33], or silicone rubber [34]. The Petri dish was positioned under a standard light microscope (at 40×) equipped with a digital video camera (Motic Moticam 2.0 MP) connected to a desktop computer (see Figure 1). Once the daphnia was in position in the video, the observer recorded a 5 s video segment of the animal, ensuring that the heartbeat was clearly captured. Thereafter, the observer recorded an additional 5 s videos every 10 min for 1 h. Immediately after the 60 min timepoint, the observer used a syringe to drip the alarm cue solution (0.1 mL) into the Petri dish. Then, we recorded an additional 5 s videos of the daphnia heartbeat every 5 min thereafter for 2 h. This completed the test procedure for the daphnia itself. Then, we captured a digital image of the daphnia for measurement, which was carried out using image analysis software provided with the camera (Motic Images Plus 3.0). We recorded the daphnia length (µm), whether it contained eggs or young, and if it was infested with *Vorticella* epibionts (see Figure 1). If epibionts were observed, we recorded the epibiont burden to be either light (less than 20 organisms), or heavy (more than 20). This entire procedure was repeated for a total of 100 individual daphnia. Lastly, we tested an additional 21 individuals (that had no *Vorticella*) to use as a ‘non-stressed control’ comparison, where each daphnia was placed in the Petri dish under the microscope, and we monitored its heart contractions as before for 3 h, except we did not add a stress treatment at all.

Following the trials, one of us reviewed the videos (in slow motion) and recorded the daphnia heart rate at each time point. With this procedure, we hoped to be able to observe changes in the daphnia heart rate before and after the addition of the alarm cue, in real time, and without any physical contact or disturbance to the daphnia.

### 2.3. Data Analysis

The objective of this study was to determine how daphnia with varying epibiont loads would react, physiologically, to the added alarm cue stressor (crushed conspecifics). The response variable of interest here was the daphnia heart rates over the 3 h monitoring period, of which there were 121 total daphnia measured separately. Visual inspection of these rates at different timepoints indicated these data were approximately normally distributed. Given that we did not have prior knowledge of the timing or duration of the reaction, if any, we conducted separate statistical tests at 5 different timepoints after the stressor was added, which we hoped would allow us to understand if, and when, the daphnia heart rate is most affected. For example, we were interested to know if the daphnia displayed an initial reaction to the stressor (i.e., after 5 min of the stress exposure), but then also if the reaction continued to grow thereafter (such as after 60 min following the stressor). For the statistical tests, we used general linear models to evaluate the effects of *Vorticella* epibionts on the heart rate reaction at each of these timepoints. Specifically, we used the 60 min heart rate (just prior to the stressor addition) as the ‘baseline’ rate for all tests and then compared how the heart rate changed from that timepoint to each other timepoint of interest (i.e., at 65, 95, 125, 155, and 180 min) by including a repeated ‘TIME’ measure in the models. We included a categorical term (‘Test group’) for the different *Vorticella* burdens on the daphnia, plus the control group, which had no stressor added (i.e., control, none, light burden, or heavy burden). Given that the daphnia under study came from the wild, they also varied in their reproductive status, where some contained eggs, or young, while others had none. We therefore included a categorical term in the analyses to account for this (eggs, young, or none). A continuous covariate to account for varying body sizes (daphnia length) was included as well. Finally, we included interaction terms involving the key parameters of interest, including the TIME*Test group interaction, which essentially tested if the heart rate response to the stressor was affected by the epibiont burdens. A copy of all raw data from this study is available in the Supplementary Materials. All analyses were conducted using the Statistica 13.3 software package (www.tibco.com).

## 3. Results

The 121 *D. ambigua* in this study ranged in size from 915 µm to 1490 µm, with an average of 1148 µm (135 µm SD). After 60 min of acclimation to the Petri dish, heart rates of the 121 *D. ambigua* ranged from 3.0 to 8.4 beats/s, with an overall mean of 5.5 beats/s. A breakdown of the average rates across all four test groups, and throughout the timepoints of interest is shown in Table S1. While it was not the goal of the study per se, we did note that daphnia with *Vorticella* epibionts (either light or heavy burdens) tended to have lower initial heart rates ($\bar{x}$ = 4.6 and 4.9 beats/s) than those that did not have epibionts (the control and the no-stress groups, $\bar{x}$ = 7.0 and 6.2 beats/s).

**Figure 2:**
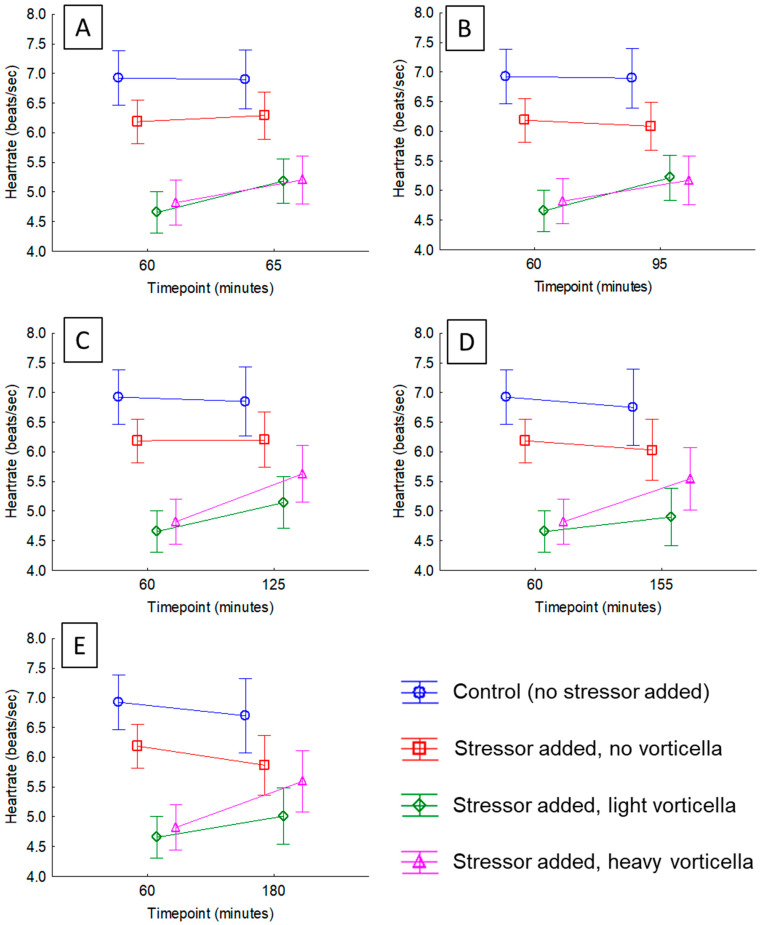
Plots of average heart rates of daphnia before and after exposure to a stressor (crushed conspecifics). The stressor was added immediately after the 60 min timepoint. The means are colored to show how the different test groups responded. In statistical analyses of these rates, the interaction term between ‘Time’ and ‘Test group’ was significant in all cases (see Tables S2–S6). Subfigures show heart rate changes after different timepoints since stress exposure, including (**A**) 5 min; (**B**) 35 min; (**C**) 65 min; (**D**) 95 min; and (**E**) 120 min.

We observed a distinct pattern after adding the crushed daphnia to the Petri dishes, which was clear no matter how we examined the data. After just 5 min following the exposure, the majority of daphnia with *Vorticella* epibionts experienced a heart rate acceleration (compared to the pre-exposure rate), but those with no *Vorticella* did not; 77% of daphnia with light epibiont loads had higher heart rates than just prior to exposure, which was different than a 50-50 distribution (ꭓ^2^ = 8.32, df = 1, *p* = 0.0.0039) and 72% of those with heavy epibiont loads had higher heart rates, which was also different than a 50-50 distribution (ꭓ^2^ = 4.56, df = 1, *p* = 0.0327). Meanwhile, only 47% of those with no Vorticella had higher heart rates, which was not significantly different than a 50-50 distribution (ꭓ^2^ = 0.095, df = 1, *p* = 0.7583). The average heart rates of each test group and across different timepoints in the project are shown in Table S1. Visual inspection of Table S1 indicates that the average heart rates of the control daphnia (no *Vorticella*, no stressor added) varied little over the 3 h monitoring period. Meanwhile, after the stressor was added, the heart rates of the daphnia in the other groups did change over time, but the magnitude of this effect depended on the epibiont load. The outcome of the general linear models for each timepoint is presented in Tables S2–S6, which also supports this conclusion, where there was a significant interaction effect at all timepoints analyzed. For example, after just 5 min of the added stressor, the interaction between ‘Time’ and ‘Test group’ is significant (*p* < 0.0001; Table S2), and this effect is visualized in Figure 2A. Heart rates of the two *Vorticella* groups increased (by about 10%), but there was no change in the group with no *Vorticella*. This same pattern continued in each of the tests thereafter (Tables S3–S6, and Figure 2B–E), where there was no meaningful change in the heart rates of daphnia with no *Vorticella*, but those that did have the epibionts had elevated heart rates after the stressor.

**Figure 3:**
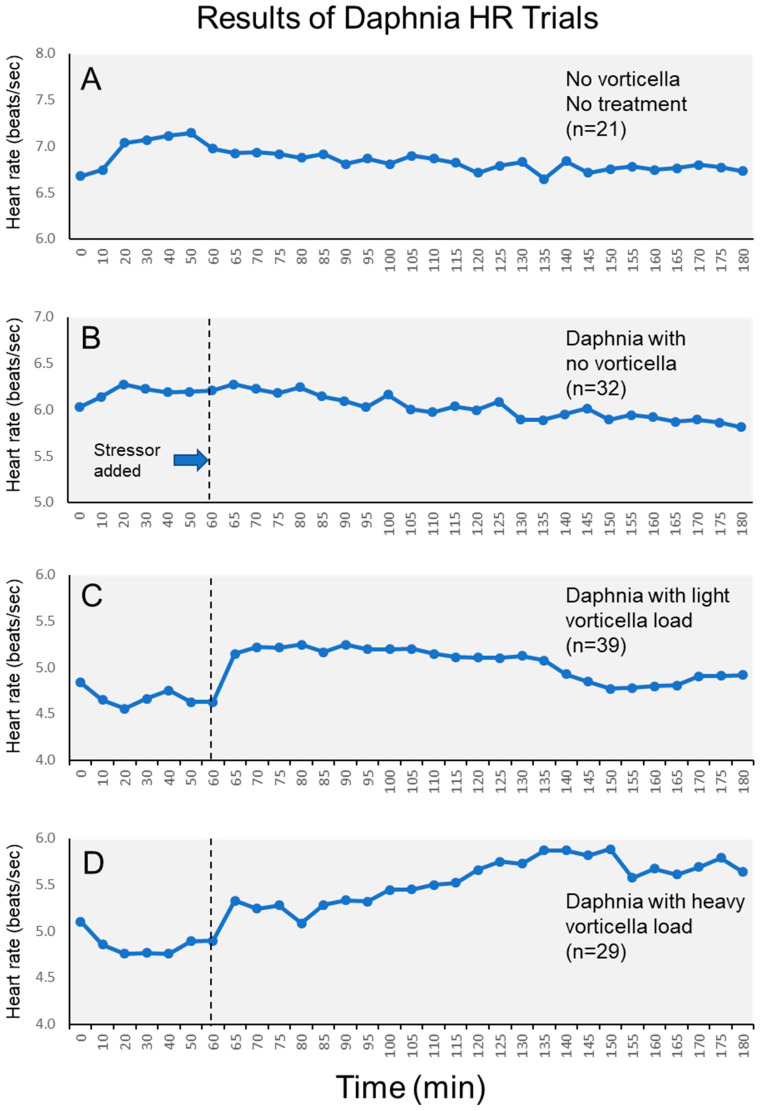
Average heart rates of daphnia across the entire 3 h monitoring period. Subfigures show different test groups of daphnia, including (**A**) control daphnia; (**B**) daphnia without *Vorticella* epibionts; (**C**) daphnia with light *Vorticella* burdens; and (**D**) daphnia with heavy *Vorticella* burdens.

The magnitude of this epibiont effect varied across the stress period, which can also be visualized in plots of the heart rates of each group across the entire 3 h period (Figure 3). Visually, we see that heart rates of daphnia with light *Vorticella* burdens tended to increase immediately after the stressor, then remained elevated for approximately 1.5 h, and then declined. Meanwhile, heart rates of daphnia with heavy *Vorticella* loads showed a pattern of continued elevation after the stressor was added. Based on the means, the heart rates of these heavily burdened daphnia were elevated by approximately 17% by one hour after the stressor was added.

Finally, while not the focus of this project, our analyses of daphnia heart rates also revealed a minor but statistically significant effect of reproductive status in some but not all timepoints during the 3 h period (Tables S2–S6). Visual inspection of the means for these three groups (at the 60 min timepoint) indicates that heart rates tended to be slightly faster when daphnia were carrying eggs than they were when they were growing young. Lastly, there was no significant heart rate variation due to daphnia body size in any of the tests (*p* > 0.3 for all, Tables S2–S6).

## 4. Discussion

Given the presumed energetic drain of heavy epibiont loads on the daphnia, we went into this project with the expectation that the *Vorticella*-infested daphnia would be *less* able to manifest a physiological stress reaction, as gauged by their heart rate response. Therefore, we expected that the daphnia with heavy epibionts would have a diminished heart rate elevation, or none at all, when the stressor was applied. We came to this idea based on other findings from the literature, showing how daphnia infected with an internal pathogen had lowered heart rates [9], and because such epibionts make it more difficult for daphnia to swim [19]. Further, research with other microorganisms has also shown how ciliated epibionts reduce fitness [18]. However, we observed the opposite pattern than what we expected, where the heaviest burdens of *Vorticella* epibionts appeared to *elevate* the heart rate response when the daphnia are exposed to a predation-mimicking stressor. This effect was not trivial, since the heart rates of heavily infested daphnia became elevated by as much as 17% after one hour of the stress exposure (Figure 3). Meanwhile, daphnia with no epibionts experienced no discernable heart rate elevation, either initially, or even after some time had elapsed (Figure 3). This pattern is statistically sound and was clear even after accounting for the minor effects of reproductive conditions on heart rates. Moreover, there was no complicating effect of daphnia size on the heart rates either.

As far as we know, this is a novel finding within the literature surrounding daphnia or other aquatic microorganisms, and so elucidating the mechanism behind this pattern will require further investigation. For now, we can at least suggest some possible avenues to explore. The first possible explanation is the simplest as well: it is possible that the ciliated *Vorticella* act to enhance the chemical signal from the crushed conspecifics by churning the water around the daphnia, and drawing more of the signal (i.e., bits of macerated tissue) toward the daphnia. In fact, we had attempted to verify if this was the case in a small follow-up experiment, though our results were inconclusive. We used a separate collection of *D. ambigua* (n = 8), which did not have any *Vorticella* burdens, and we exposed each to the same stress procedures, including 3 h of heart rate monitoring, exposure to the crushed daphnia alarm cue, and, only in these trials, one of us attempted to stir the water in the Petri dish to ensure the animals were receiving the cue; this was performed using a plastic syringe, and the observer swished the water in circles around the Vaseline-glued daphnia for 10 s just prior to the video recordings. Comparison of heart rates before and after the stress exposure showed no difference after 5 min (paired *t*-test, *t* = 1.11, *p* = 0.3042), no difference after 30 min (*t* = 0.25, *p* = 0.8089), but a slight elevation after 60 min (*t* = 2.49, *p* = 0.0411) and 90 min (*t* = 2.37, *p* = 0.0498), but not after 2 h (*t* = 2.23, *p* = 0.3041). These findings could be interpreted as providing partial support for the idea that the *Vorticella* act to mix the water around the daphnia, thereby drawing the alarm cue closer.

Another possibility is that the *Vorticella* actually amplify the chemical alarm cues around the daphnia by further breaking down (digesting them) the bits of tissues from the crushed conspecifics in the water, which may release more of the alarm cue chemicals from the tissues. *Vorticella* eat by drawing food into their bell (using the beating cilia on their bell mouth), and then excreting waste material following digestion. Digestion times of some species of ciliates are between 60 and 90 min [35], which is consistent with the timing of the heartbeat acceleration in our study. A third possibility is that the presence of *Vorticella* on daphnia, especially over the life of the daphnia, somehow “predisposes” those daphnia to a rapid and sustained physiological response to threats. Perhaps by depriving the host daphnia of food [17], and over its lifetime, this creates a ‘chronic stress’ condition in the hosts, which makes them react more strongly to acute stressors.

The fact that the heavily burdened daphnia showed a pronounced and continued heart rate elevation (Figure 3) after the initial stressor could have an alternative explanation beyond simply a “stress” response. Since we did not change the water in the Petri dishes during the 3 h experiment, the presence of so many *Vorticella* organisms could have slowly reduced the overall oxygen concentration in the dish [36], which itself could cause a compensatory elevation in the daphnia metabolism, with a corresponding elevation of heart rate [37]. To further elucidate this, additional experiments would be required wherein the daphnia would be maintained in larger water containers, or those with the continuous addition of oxygen.

In terms of interpreting our findings, there is a final possibility that bears mentioning, which relates to the fact that our project focused on wild daphnia that naturally varied in epibiont loads. Since we did not experimentally manipulate the epibionts in the project, our results are based on comparisons of daphnia without *Vorticella* epibionts to those that did have these upon capture. We have inferred that the patterns we observed in heart rates between infested and non-infested daphnia are indeed the effects of the epibionts. However, it is remotely possible that these two groups of daphnia have inherently different physiologies, or stress responses, that may not be related to the epibionts, per se. Consider that the daphnia with no *Vorticella* tended to have faster heart rates to begin with (Figure 2, Table S1). This could imply that these individuals have a higher metabolism [25,37], which in turn means they might be more physically active, and perhaps less likely to become colonized by epibionts in the first place. The fact that their metabolism (heart rate) is high to begin with could then preclude their heart from becoming further accelerated when stressed. A test of this idea could involve manipulating *Vorticella* burdens on naïve daphnia, whereby the animals would be first tested without any epibionts, and then tested again after receiving epibionts. Or, daphnia with epibionts could be tested, and then their epibionts could be somehow removed, and the animals tested again. Both of these scenarios would require a degree of skill in the micro-manipulation of the stalked ciliates.

A robust cardiac reaction to a stressor should be of importance when animals need to temporarily enhance their muscle function, perhaps to escape a predator. It would therefore be interesting to conduct follow-up tests of this idea using daphnia, and to ask if the *Vorticella*-infested individuals actually do have an enhanced “escape response”, or in other words, if they actually exert more energy when they become stressed, compared to non-infested daphnia. However, one must also consider how the heavy burdens of epibionts may actually impede swimming performance because of the increased drag. One wonders, then, if the heightened cardiac stress reactions of such daphnia are the result of living with such burdens over long time periods, and having to exert more energy on demand (just to swim away) whenever a predator is perceived.

While it was not the focus of the project, we noted an interesting pattern in the daphnia heart rates during the initial “acclimation” period of the monitoring, immediately after the animals were secured to the Petri dishes. This pattern is clear from Figure 3, where the daphnia without *Vorticella* experienced a mild elevation in heart rate within the first 20 min, while those with *Vorticella* experienced a mild depression of heart rates in the same timeframe. Interestingly, these patterns are the opposite of what we observed following the simulated predation stressor at the 60 min mark. Certainly, changes in heart rates would be expected following the brief physical manipulation of the animals (which they likely perceive as stressful), though the fact that the directionality of these changes depended on the presence of epibionts is another sign of how the daphnia stress responses are sensitive to change, and, that they can be affected in different ways by a different type of stressors.

There has been a long history of investigations into how heart rates of daphnia are affected by various chemical or environmental stressors [9,33,34,38,39,40], though the majority of these studies involve comparing heart rates of different groups of daphnia after lengthy, chronic exposures to the stressor in question (days, for example), or comparisons of different groups of daphnia under different exposures of stressors. These studies are worthwhile, though our study highlights how the acute stress response can be meaningful to study within individual daphnia, over shorter intervals (minutes). This allows for the identification and quantification of the magnitude of the cardiac stress reaction, plus the duration of the response, as in, how long the heart rate elevation lasts (see Figure 3). To be fair, the cardiac response to stressors is only one facet of the overall stress reaction of an organism, both in invertebrates and vertebrates, which also can include changes to respiration, hormonal or neurochemical changes, immune function modification, and/or behavioral responses [2,6,41,42,43,44,45]. For some research with vertebrates, the magnitude and duration of this physiological reaction are valuable predictors of the overall health and future well-being of the animals [46,47,48]. Our study therefore provides a useful starting point for such work within the realm of the microorganisms.

## Data Availability

The original contributions presented in the study are included in the article/Supplementary Materials, further inquiries can be directed to the corresponding author.

## Supplementary Materials

The following supporting information can be downloaded at: https://www.mdpi.com/article/10.3390/microorganisms12061219/s1, Figure S1: additional images of *Daphnia ambigua* with and without *Vorticella* epibionts; Table S1: average heart rates (beats/s) of daphnia from each test group at key timepoints in the experiment. The 60 min mark was used as the initial (acclimated) heart rate, and then the alarm cue was added; Table S2: GLM examining the initial HR reaction to the alarm cue (comparing time 60 to time 65); Table S3: GLM examining HR reaction after 30 min (time 60 to time 95); Table S4: GLM examining HR reaction after 60 min (time 60 to time 125); Table S5: GLM examining HR reaction after 90 min (time 60 to time 155); Table S6: GLM examining HR reaction after 120 min (time 60 to time 180).
