# Supplementary material for: Ciliated Epibionts Modify the Cardiac Stress Reaction to Perceived Predation in Daphnia
Source: Microorganisms. 2024 Jun 18;12(6):1219. doi: 10.3390/microorganisms12061219 (PMC11205733; doi:10.3390/microorganisms12061219)
Supplement: Supplementary file 1 [file microorganisms-12-01219-s001.zip › microorganisms-3043706-supplementary.pdf]

Supplemental file for Davis and Gloege

Ciliated Epibionts Modify the Cardiac Stress Reaction to Perceived Predation in Daphnia

The following document contains supplemental material related to the Davis and Gloege study, including:

- Figure S1 – additional images of *Daphnia ambigua* with and without *Vorticella* epibionts

- Table S1 – average heart rates of each daphnia test group across different timepoints after the stressor was added

- Table S2 – Summary of statistical tests that evaluated how the heart rates vary with each predictor

Figure S1

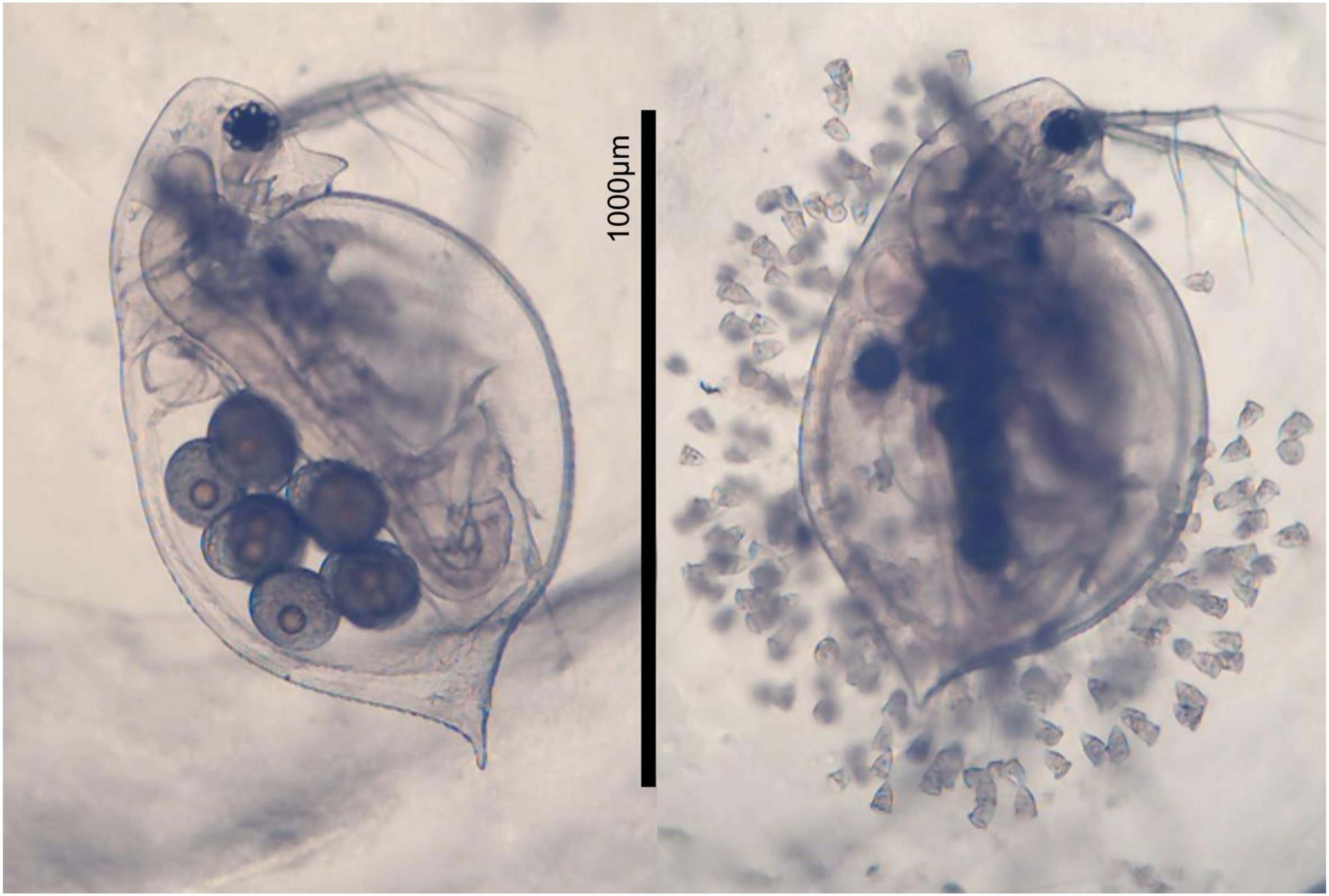

Table S1. Average heart rates (beats/sec) of daphnia from each test group at key timepoints in the experiment. The 60-minute mark was used as the initial (acclimated) heart rate, then the alarm cue was added.

**Daphnia Test Group**	**N**	**HR at 60min**	**HR at 65min**	**HR at 95min**	**HR at 125min**	**HR at 155min**	**HR at 180min**
Control (no *Vorticella*, no stressor)	21	6.97	6.92	6.87	6.79	6.78	6.73
Stressor added, no *Vorticella*	32	6.21	6.28	6.03	6.09	5.94	5.81
Stressor added, light *Vorticella*	39	4.63	5.15	5.19	5.10	4.78	4.92
Stressor added, heavy *Vorticella*	29	4.90	5.32	5.32	5.74	5.57	5.63
All Daphnia	121	5.52	5.80	5.73	5.81	5.62	5.64

Tables S2-S6. Summary of statistical analyses of daphnia heart rates. These tests were designed to elucidate the effect of an alarm cue (crushed conspecifics) on heart rates of daphnia that have different burdens of epibionts (ciliated *Vorticella* spp.). Five separate GLM analyses were conducted (corresponding to different timepoints), with each test having the same predictor variables. Daphnia were allowed to acclimate for 60 minutes prior to the exposure, and we used their 60-minute heart rate as the control rate for each test. Each test below therefore examined how the heart rate changed from the 60-minute timepoint to the later timepoint ('TIME' was a repeated measure in the analysis). There were 4 experimental groups of daphnia, including 1) control (no epibionts, no alarm cue), 2) daphnia with no epibionts, 3) daphnia with light epibiont burden, and 4) daphnia with heavy epibiont burden. Each test also included a categorical factor to account for the varying reproductive conditions (daphnia with eggs, daphnia with young, or daphnia with nothing), as well as a continuous covariate to account for daphnia size (length). The factor of primary interest to us was the Time*Test Group interaction (highlighted).

Table S2. GLM examining the initial HR reaction to the alarm cue (comparing time 60 to time 65)

Predictor	SS	df	MS	F	p
Daphnia length	1.03	1	1.03	0.51	0.47702
Daphnia Test Group	119.48	3	39.83	19.63	0.00000
Reproductive state	12.59	2	6.29	3.10	0.04878
Error	231.27	114	2.03		
TIME	0.02	1	0.02	0.21	0.65111
TIME*Daphnia length	0.00	1	0.00	0.01	0.91472
**TIME*Test Group**	**2.19**	**3**	**0.73**	**6.41**	**0.00047**
TIME*Reproductive state	0.51	2	0.25	2.24	0.11111
Error	12.96	114	0.11		

Table S3. GLM examining HR reaction after 30min (time 60 to time 95)

Predictor	SS	df	MS	F	p
Daphnia length	0.56	1	0.56	0.27	0.60328
Daphnia Test Group	113.44	3	37.81	18.38	0.00000
Reproductive state	14.32	2	7.16	3.48	0.03408
Error	234.48	114	2.06		
TIME	0.14	1	0.14	1.31	0.25453
TIME*Daphnia length	0.05	1	0.05	0.49	0.48388
**TIME*Test Group**	**3.83**	**3**	**1.28**	**11.64**	**0.00000**
TIME*Reproductive state	1.24	2	0.62	5.64	0.00463
Error	12.50	114	0.11		

Table S4. GLM examining HR reaction after 60min (time 60 to time 125)

Predictor	SS	df	MS	F	p
Daphnia length	0.49	1	0.49	0.21	0.64562
Daphnia Test Group	105.46	3	35.15	15.27	0.00000
Reproductive state	9.74	2	4.87	2.11	0.12535
Error	262.41	114	2.30		
TIME	0.26	1	0.26	0.95	0.33157
TIME*Daphnia length	0.08	1	0.08	0.28	0.59528
**TIME*Test Group**	**5.86**	**3**	**1.95**	**7.05**	**0.00022**
TIME*Reproductive state	2.11	2	1.05	3.80	0.02531
Error	31.60	114	0.28		

Table S5. GLM examining HR reaction after 90min (time 60 to time 155)

Predictor	SS	df	MS	F	p
Daphnia length	1.93	1	1.93	0.85	0.35885
Daphnia Test Group	109.60	3	36.53	16.04	0.00000
Reproductive state	3.36	2	1.68	0.74	0.48040
Error	259.63	114	2.28		
TIME	0.08	1	0.08	0.12	0.72527
TIME*Daphnia length	0.17	1	0.17	0.26	0.61164
**TIME*Test Group**	**6.41**	**3**	**2.14**	**3.29**	**0.02329**
TIME*Reproductive state	5.43	2	2.71	4.18	0.01776
Error	74.04	114	0.65		

Table S6. GLM examining HR reaction after 120min (time 60 to time 180)

Predictor	SS	df	MS	F	p
Daphnia length	1.57	1	1.57	0.74	0.39204
Daphnia Test Group	97.82	3	32.61	15.34	0.00000
Reproductive state	3.88	2	1.94	0.91	0.40420
Error	242.31	114	2.13		
TIME	0.03	1	0.03	0.04	0.85017
TIME*Daphnia length	0.07	1	0.07	0.10	0.74718
**TIME*Test Group**	**9.57**	**3**	**3.19**	**4.48**	**0.00517**
TIME*Reproductive state	2.50	2	1.25	1.76	0.17741
Error	81.14	114	0.71		
